# Improvising in Endoscopy: Endoscopic Removal of Sharp Foreign Bodies in the Upper GI Tract, Using a Handmade Protective Device

**DOI:** 10.1155/2020/8881702

**Published:** 2020-09-09

**Authors:** Konstantinos Miltiadou, Zisimangelos Solomos, Dimitrios Lygkos, Alexandros Chatzidakis, Evripidis Rizos, Ioannis S. Papanikolaou, Konstantinos Triantafyllou

**Affiliations:** ^1^Hepatogastroenterology Unit, 2^nd^ Department of Internal Medicine-Propaedeutic, Research Institute and Diabetes Center, Medical School, National and Kapodistrian University, Attikon University General Hospital, Athens 124 62, Greece; ^2^Doctors of the World, Greek Delegation, Athens 105 53, Greece

## Abstract

*Introduction.* Foreign body ingestion is a common problem in large-volume endoscopic departments. Several techniques and devices have been described for the safe endoscopic removal of these objects. However, these devices may not be suitable in every clinical setting or—as in our case—they may not even be available. *Case Presentation.* We report the case of a 34-year-old patient, presenting with sharp foreign body ingestion. The foreign bodies were safely removed using a handmade protective hood due to lack of a commercial device. In our case, improvisation proved to be of great benefit for the patient as well as for the endoscopist. *Discussion.* Improvised interventions can be of special interest in the setting of insufficiently funded or equipped endoscopic departments.

## 1. Introduction

Ingestion of foreign bodies is a common problem in large-volume endoscopic departments. In adults, it is usually related to elderly, psychosocial-developmental problems, intoxication, and incarceration. Although up to 80% of them will pass spontaneously, the ingestion of sharp foreign bodies can be technically challenging for the endoscopist and dangerous for the patient, because the risk of complications (esophagogastric/pharyngeal damage and aspiration) is as high as 35%, especially if not removed in time [1–4]. Therefore, several techniques have been proposed [5–12] and the use of protective devices is recommended, to avoid these complications during extraction of sharp foreign bodies [4].

A variety of protective devices are available to safely aid endoscopic removal of such objects [13]. However, these devices may not be suitable in every clinical setting [5] and sometimes, they may not even be available. In such occasions, a case-by-case approach or even improvisation can be beneficial for the patient (and perhaps the endoscopist). Examples of such handmade devices (caps, condoms, gloves, and tubes) have been reported in the literature [14–18].

Herein, we present a case of sharp foreign bodies removed from the stomach of a patient, using a handmade rubber hood, fashioned from a plastic transfusion pressure infusor.

## 2. Case Presentation

**Figure 1:**
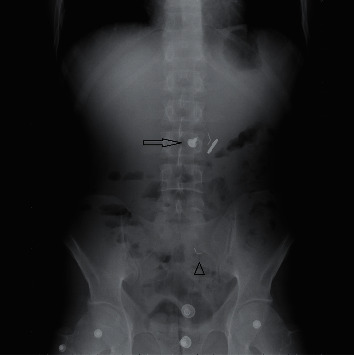
Abdominal X-ray: arrow showing 3 metallic objects (screw, coin, and plastic head pin) in the stomach, and arrow head showing another plastic head pin well into the small intestine. Note that the small metal scrap that was later seen on endoscopy is not evident here.

A 34-year-old male patient with no prior medical history presented at the Emergency Department reporting ingestion of several metallic objects one hour before. The clinical examination was unremarkable, and the patient was in overall excellent condition. X-ray studies revealed at least 4 metallic objects in various parts of the GI tract (Figure 1).

**Figure 2:**
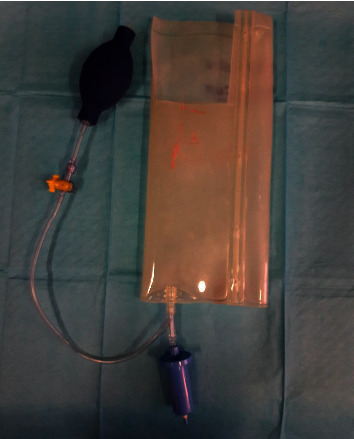
A plastic transfusion pressure infusor. Note the missing rubber part used to construct the hood.

**Figure 3:**
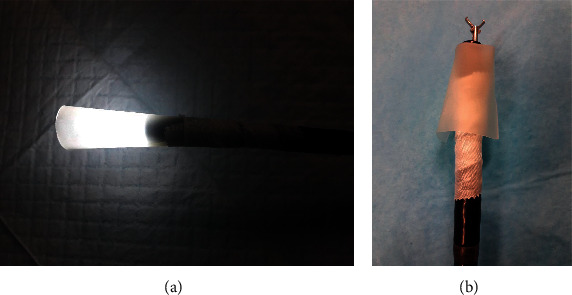
(a) Rubber folded in coned fashion forming a bell-shaped hood and secured with silk tape. (b) Hood-inverted back on itself before scope insertion.

**Figure 4:**
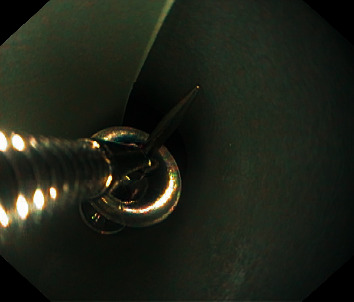
Testing if the foreign body fits in the hood before inserting the scope.

Considering the timing of ingestion and the type of the ingested materials, endoscopic extraction was decided. Esophagogastroduodenoscopy (EGD) was performed approximately 2 hours after the ingestion. Four objects were visualized at the time of endoscopy (one screw in the first part of the duodenum, one elongated pin impacted in the antrum wall, one coin, and one small metal scrap in the stomach). The coin was easily removed from the stomach using a Roth Net. An overtube was then introduced in order to safely retrieve the remaining 2 larger sharp objects. None of the remaining objects could fit in the overtube, and the patient's tolerance to the procedure was poor despite maximal sedation. No other protective devices such as rubber hoods were available at the time of endoscopy. Therefore, any further efforts to extract the objects were abandoned, and the patient was returned to the surgical ward for monitoring. Recognizing that complication rates could be high in this patient and that referral to another better equipped facility would require time, such that it would expose the patient to the risks of delayed intervention, we improvised a handmade rubber hood, constructed from a plastic transfusion pressure infusor (Figure 2). This material was chosen because it was flexible enough to invert back on itself in the cardia upon withdrawal of the scope, yet thick enough to withstand puncturing. The hood was fastened with silk tape to the distal end of a 9.8 mm gastroscope (GIF-Q165 Olympus Corporation, Tokyo, Japan), in a coned fashion (to facilitate the entrance of the foreign body into the bell of the hood), and then inverted (Figures 3(a) and 3(b)). A test was carried out before scope insertion to make sure that the foreign body fits in the hood (Figure 4).

**Figure 5:**
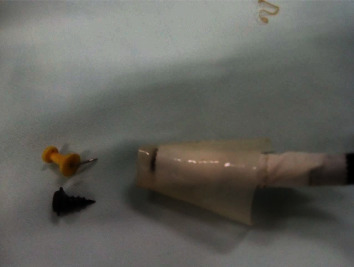
Elongated pin and thick screw safely extracted. Note the bended tip of the pin which was impacted in the antrum wall.

The patient was called for the second EGD three hours after the first one. The two larger foreign bodies were still in the stomach, whilst the small metal scrap had migrated distally. With the help of mild sedation (3 mg of midazolam), the pin and the screw were captured using rat-tooth forceps, pulled into the hood, and extracted (Figure 5). The hood inverted back on itself as expected, with no tissue injury upon second endoscopic look. The procedure lasted only 15 minutes with excellent tolerance from the patient.

The patient was returned to the ward for monitoring because two sharp objects were still in his small bowel. Repeat X-ray studies the following days confirmed successful discharge of the foreign bodies per rectum without any complications.

## 3. Discussion

We have presented a case of successful endoscopic sharp foreign body extraction, using a handmade protective hood. To our knowledge, this is the first time that the specific device has been used, although examples of similar handmade devices (caps, condoms, gloves, and tubes) have been successfully used and reported in the literature [14–18]. In our case, it was very helpful in a moment of need, when a commercial protective hood was unavailable. It proved beneficial for both the patient and the frustrated endoscopist.

This scenario could also be relevant in the setting of small, remote, inadequately equipped or funded endoscopic departments, especially if an anesthesiologist is not readily available to intubate or heavily sedate the young patient who cannot tolerate large overtubes.

The device seems to offer some advantages: it practically comes at no cost; it can be constructed in a matter of minutes; it offers good visualization of the lumen; and it seems to be safe. On the other hand, it is a handmade, nonvalidated device which cannot be duplicated in the exact same form, and therefore, its performance is unpredictable. There are concerns that the device may not invert in the cardia, or even dislodge from the endoscope, adding one more foreign body in the stomach. However, if one is found in a situation of limited options such as ours, it seems relatively safe to give it a try and abandon the extraction if the device does not fold properly, or easily extract the device with a Roth Net if dislodged in the stomach.

## 4. Conclusion

In our case, improvisation proved to be of great benefit for the patient as well as for the endoscopist. Such interventions can be of special interest in the setting of insufficiently funded or equipped endoscopic departments.

## Data Availability

Data of the case are available as figures in the manuscript.
